# Omentin-1 Stimulates Human Osteoblast Proliferation through PI3K/Akt Signal Pathway

**DOI:** 10.1155/2013/368970

**Published:** 2013-03-31

**Authors:** Shan-Shan Wu, Qiu-Hua Liang, Yuan Liu, Rong-Rong Cui, Ling-Qing Yuan, Er-Yuan Liao

**Affiliations:** Institute of Metabolism and Endocrinology, Second Xiang-Ya Hospital, Central South University, Changsha 410011, Hunan, China

## Abstract

It has been presumed that adipokines deriving from adipose tissue may play important roles in bone metabolism. Omentin-1, a novel adipokine, which is selectively expressed in visceral adipose tissue, has been reported to stimulate proliferation and inhibit differentiation of mouse osteoblast. However, little information refers to the effect of omentin-1 on human osteoblast (hOB) proliferation. The current study examined the potential effects of omentin-1 on proliferation in hOB and the signal pathway involved. Omentin-1 promoted hOB proliferation in a dose-dependent manner as determined by [^3^H]thymidine incorporation. Western blot analysis revealed that omentin-1 induced activation of Akt (phosphatidylinositol-3 kinase downstream effector) and such effect was impeded by transfection of hOB with Akt-siRNA. Furthermore, LY294002 (a selective PI3K inhibitor) and HIMO (a selective Akt inhibitor) abolished the omentin-1-induced hOB proliferation. These findings indicate that omentin-1 induces hOB proliferation via the PI3K/Akt signaling pathway and suggest that osteoblast is a direct target of omentin-1.

## 1. Introduction

Adipokines, secreted by adipose tissue, have been demonstrated to play critical roles in regulating metabolic homeostasis, insulin sensitivity, systemic inflammatory processes, cardiovascular function, and bone metabolism [1, 2]. Recently, adipokines have emerged as elements in the regulation of bone metabolism [3, 4]. Previous studies proved that the adipokines such as leptin and adiponectin could modulate bone metabolism both *in vitro* and *in vivo* [5–9]. Our previous work showed that apelin and vaspin inhibited the apoptosis of human osteoblast (hOB) [10, 11], and adiponectin stimulated proliferation and differentiation of hOB [12]. However, the function and mechanism involved await to be elucidated.

Omentin-1, also named intelectin-1, is a newly discovered 34 kDa adipokine selectively expressed in omental adipose tissue and abundantly present in plasma [13, 14]. Omentin-1 participates in multiple physiological processes including insulin action, cardiovascular function, and inflammatory response. It was reported that omentin-1 could modulate insulin sensitivity [14], inhibit TNF-induced vascular inflammation in human endothelial cells [15], and induce vasodilation [16]. Recent study demonstrated that omentin-1 played a protective role against vascular calcification [17]. Clinical studies showed that omentin-1 levels inversely correlated with obesity and insulin resistance [18]. Regarding its effects on bone, recent study reported that circulating omentin-1 levels had an inverse correlation with bone mineral density (BMD) at lumber spine in Iranian postmenopausal women [19]. Xie et al.'s study demonstrated that omentin-1 could alleviate the bone loss in osteoprotegerin-deficient or ovariectomized mice by regulating the proliferation and differentiation of the mouse osteoblast [20, 21]. However, research concerning the potential effects of omentin-1 on hOB proliferation remains relatively poor. Our present work focuses on the role of omentin-1 in controlling hOB proliferation and the signaling pathway involved.

## 2. Materials and Methods

### 2.1. Reagents

Recombinant omentin-1 was the product of Cell Science, Inc. (Canton, MA, USA). Anti-Akt and p-Akt antibodies, anti-mouse, and rabbit IgG peroxidase conjugate antibodies were purchased from Santa Cruz Biotechnology Inc. (Waltham, MA, USA). LY294002 and HIMO were purchased from Calbiochem Corp. (San Diego, CA, USA). 

### 2.2. Cell Cultures

Primary hOB was isolated from human trabecular bone obtained during surgery following traffic accident victims as previously described [22, 23], and after being approved by the Ethics Committee of the Second Xiangya Hospital of Central South University, China. None of the donors suffered from clinical symptoms or history of bone metabolic disorders. Briefly, samples were washed extensively with phosphate buffered saline (PBS) to remove blood cells and debris and finally washed in culture medium. Then, the sample was digested with type IV collagenase (Sigma) and cultured in phenol red-free *α*-MEM containing 10% fetal bovine serum (FBS, Gibco-BRL Corp. Grand Island, NY, USA), 100 U/mL penicillin, 100 *μ*g/mL streptomycin, and 50 *μ*g/mL ascorbic acid (Sigma) at 37°C in a humidified incubator with 5% CO_2_. Medium was changed every 2 days and after approximately 4 weeks in culture, cells were harvested using trypsin EDTA and subcultured in *α*-MEM containing 10% FBS, 100 U/mL penicillin, 100 *μ*g/mL streptomycin, and 50 *μ*g/mL ascorbic acid. Osteoblast was cultured to facilitate mineralization in differentiation medium containing 10% FBS, 50 *μ*g/mL ascorbic acid, 10 nM dexamethasone, and 10 mM *β*-glycerophosphate. The phenotype of cells was characterized based on the ALP activity, osteocalcin (OC) secretion, and the formation of mineralization nodules as previously described [22, 23]. Briefly, ALP activity was assayed by spectrophotometric measurement of p-nitrophenol release at 37°C. ALP activity was normalized to total protein content of the cell layer. Osteocalcin released into the culture media was measured using a specific radioimmunoassay kit (DiaSorin, Stillwater, MN, USA). To normalize protein expression to total cellular protein, a fraction of the lysate solution was used in a Bradford protein assay. The formation of mineralization nodules was determined by Alizarin Red S staining.

### 2.3. Assessment of Cell Proliferation

HOB proliferation was assessed using [^3^H]thymidine (2 mCi/mL) incorporation into trichloroacetic acid (TCA) insoluble material followed by scintillation counting. Briefly, cells were plated at a density of 2 × 10^4^ cells/well in 24-well plates and treated with 25–200 ng/mL omentin-1 for 48 h, in the presence of [^3^H]thymidine. 24 hours later, the plates were washed with PBS, and 10% TCA solution was added to the wells. Incorporated [^3^H]thymidine was released by washing with 0.2 N of NaOH, and radioactivity was measured using a *β*-scintillation counter. Results are expressed as counts per minute.

To study the effects of inhibitors, cells were pretreated with PI3K inhibitor LY294002 (10 *μ*M), or Akt inhibitor HIMO (10 *μ*M) for 3 h prior to treatment with 200 ng/mL omentin-1.

### 2.4. Detection of Akt Activation

Briefly, hOBs were first treated with 200 ng/mL omentin-1 for 5–60 min. Then, cell monolayers were washed quickly with cold PBS containing 5 mM of EDTA and 0.1 mM of Na_3_VO_4_ and lysed with a lysis buffer consisting of 20 mM of Tris-HCl (pH 7.5), 150 mM of NaCl, 1% Triton X-100, 10 mM of NaH_2_PO_4_, 10% glycerol, 2 mM of Na_3_VO_4_, 10 mM of NaF, 1 mM of ABSF, 10 mg/mL leupeptin, and 10 mg/mL aprotinin. Protein concentrations were determined by Bradford assay. 10 *μ*g of protein was loaded onto a 10% polyacrylamide gel. After electrophoresis, the SDS-PAGE separated proteins were transferred to a nitrocellulose membrane (Amersham Pharmacia Biotech). The membrane was blocked with 2.5% nonfat milk in PBS and incubated with anti-Akt and -phospho-Akt primary antibodies (Santa Cruz, Biotechnology, CA, USA) at 1 : 500 in PBS for 2 h. Then, the membrane was incubated with goat anti-mouse or rabbit IgG conjugated with horseradish peroxidase (Santa Cruz) at 1 : 1000 in PBS for 1 h. Blots were processed using an ECL (Santa Cruz) kit and exposed to X-ray film.

### 2.5. Genetic Suppression of Akt by siRNA

For gene knockdown experiments, hOBs were plated in 60 mm diameter dish and cultured for 24 h in medium without antibiotics. To suppress Akt, the hOBs were transfected with either Akt small interfering RNA (siRNA) or control siRNA (Santa Cruz Biotechnology Inc.) using Lipofectamine 2000 (Invitrogen, Carlsbad, CA, USA). The levels of Akt expression were analyzed by western blotting as described above.

### 2.6. Statistical Analyses

Data are presented as the mean ± SD. Comparisons were made using a one-way ANOVA. All experiments were repeated at least three times, and representative experiments are shown.

## 3. Results

### 3.1. Characterization of hOB

Cells were identified as osteoblast using several criteria, including high intrinsic ALP activity, secretion of OC, and mineralized nodule formation as previously described [24]. The ALP activities in normal hOB were 71.6 ± 6.3 nmol/min/mg protein. The OC levels in the culture supernatants from unstimulated human bone cells were 4.65 ± 0.36 ng/mg protein. After 21 days of culture with differentiation medium, the mineralized nodule formation was detected using Alizarin Red S staining in cultured hOB. Fulfilling the above criteria for osteoblast, our results demonstrated that the cells isolated were primary hOB from collagenase-digested human trabecular bone.

### 3.2. Omentin-1 Stimulated hOB Proliferation

**Figure 1:**
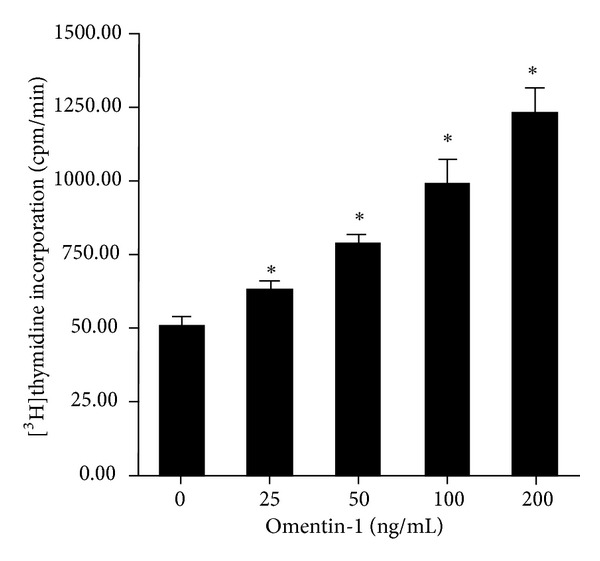
Omentin-1 stimulated the proliferation of hOB. Cells were exposed to 25–200 ng/mL omentin-1 for 48 h. Cell proliferation was determined by measuring [^3^H]thymidine incorporation. Results are expressed as counts per minute. *P < 0.05 versus control.

Using [^3^H]thymidine incorporation by cells to determine the proliferation of hOB, we confirmed that omentin-1 stimulated hOB proliferation in a dose-dependent manner. Compared to the control group, cells treated with omentin-1 at concentrations of 25, 50, 100, and 200 ng/mL increased the [^3^H]thymidine incorporation of hOB by 23.66%, 53.29%, 93.93%, and 140.36%, respectively, with statistical significance (all *P* < 0.05) (Figure 1). 

### 3.3. Omentin-1 Activated Akt Signaling Pathway in hOB

**Figure 2:**
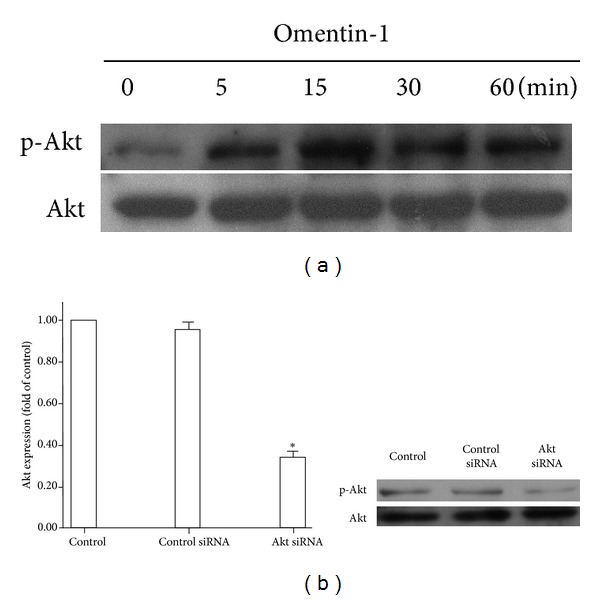
Omentin-1 activated Akt signal pathway in hOB. (a) Western blot analysis of Akt activation. The hOBs were cultured in serum-free a-MEM for 6 h and then treated with omentin-1 (200 ng/mL) for 5–60 min. The cell lysates were analyzed by western blotting and incubated with antibodies against p-Akt and Akt. Representative results are shown. (b) Either control siRNA or Akt siRNA was transfected into osteoblast. Akt siRNA significantly suppressed the expression of phosphor-Akt (left). Expression of Akt was determined by western blot analysis using an Akt antibody. Representative results are shown (right).

To investigate the signal pathway involving omentin-1, we determined if the Akt signaling pathway was inducible by omentin-1. As shown in Figure 2(a), omentin-1 stimulated the activity of Akt in hOB after 5 min incubation with omentin-1 as demonstrated by an increased phosphorylated Akt levels. 

To determine the effect of Akt in the proliferation of omentin-1 on hOB, we used siRNA to knockdown the expression of Akt. As shown in Figure 2(b), transfection of hOB with Akt siRNA inhibited Akt protein expression.

### 3.4. Omentin-1 Regulated Proliferation of hOB through the PI3K/Akt Signaling Pathway

**Figure 3:**
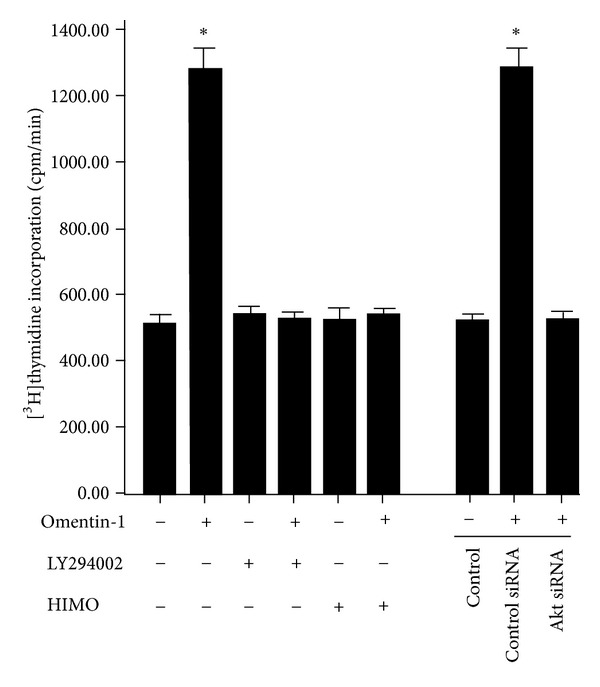
Omentin-1 regulated proliferation of hOB through the PI3K/Akt signaling pathway. The hOBs were pretreated with vehicle, PI3K inhibitor LY294002 (10 *μ*M), or Akt inhibitor HIMO (10 *μ*M) for 3 h prior to treatment with omentin-1 (200 ng/mL) for 48 h. The cells were also transfected with control siRNA or Akt siRNA before treatment with omentin-1 (200 ng/mL) for 48 h. Cell proliferation was determined by measuring [^3^H]thymidine incorporation. Results are expressed as counts per minute. *P < 0.05 versus control.

Because the results above demonstrated that omentin-1 activated Akt signaling pathway in hOB, we examined whether the omentin-1-induced proliferation is mediated via the activation of PI3K/Akt signaling pathway. Pretreatment of cells with the PI3K inhibitor LY294002 or Akt inhibitor HIMO abolished the omentin-1-induced cell proliferation (Figure 3). The observation from Akt siRNA treatment cohered with the current observation when cells are treated with LY294002 and HIMO. In conclusion, treatment of hOB with Akt siRNA suppressed the effects of omentin-1 on proliferation in hOB (Figure 3).

## 4. Discussion

The present study shows that treatment with omentin-1 stimulates proliferation of hOB, indicating a growth promotion effect of omentin-1 on hOB. It is also shown that PI3K/Akt signal pathway is a key mediator of such effect.

Adipose tissue has been recognized as a highly active endocrine organ. In addition to the uptake, storage, and synthesis of lipids, adipose tissue secrets a variety of adipokines (e.g., adiponectin, leptin, resistin, vaspin, and visfatin). These adipokines control insulin sensitivity, neuroendocrine activity, food and water intake, breeding, inflammatory response, cardiovascular function, and bone metabolism [1, 21]. Omentin-1, a new adipokine, is primarily expressed in visceral adipose tissue and abundant in plasma [13, 14]. Previous studies have demonstrated that both omentin-1 mRNA and plasma levels inversely correlated to obesity, BMI, and insulin resistance [18, 25], and serum omentin-1 was found decreased in diabetic patients [26, 27]. Furthermore, omentin-1 plays critical roles in cardiovascular protecting, such as inducing vasodilation, inhibiting osteoblastic differentiation of vascular smooth muscle cells (VSMCs) [16, 17]. It has been noted that those effects of omentin-1 is similar to cardiovascular protecting adipokine adiponectin, an insulin sensitizer [28–30]. Our previous study has shown that adiponectin stimulated the proliferation and differentiation of hOB [12]. Several other adipokines such as leptin, resistin, and visfatin had been identified to induce the proliferation of osteoblast [31–33], suggesting a close relationship between adipokine and osteoblast. Clinical investigations have demonstrated that serum omentin-1 negatively correlated with BMD in the anorexia nervosa girls [34] and Iranian postmenopausal women [19]. Xie et al. have also found that omentin-1 ameliorated bone loss of ovary ectomized mice and OPG^−/−^ mice, and that omentin-1 stimulated proliferation and inhibited differentiation of mouse primary osteoblast [20, 21]. 

In the present experiment, 25–200 ng/mL omentin-1 has been chosen to treat the hOB based on the previous research [21]. To determine the effect of omentin-1 on hOB proliferation, [^3^H]thymidine incorporation assay was adopted. Our data have demonstrated that omentin-1 stimulated the proliferation of hOB in a dose-dependent manner. The proliferative effect of omentin-1 on hOB is therefore consistent with the report by Xie et al. [21]. 

To gain further insight into the underlying mechanism about how omentin-1 stimulated hOB proliferation, we evaluated signaling pathway that was potentially involved. Multiple signaling pathways, such as MAPK, Wnt, AMP, and PI3K-Akt, were found to participate in the modulation of osteoblast proliferation [35–38]. Among this, PI3K-Akt, which existed in all mammalian cells and exerted profound effects on diverse processes including cell proliferation, survival, differentiation, migration, and metabolism, was the most important one. Our study showed that omentin-1 induced the activation of Akt in hOB, whereas knocking down the expression of Akt with siRNA impeded the stimulatory effect of omentin-1 in hOB proliferation. In addition, pretreatment of hOB with LY294002 or HIMO could also block the effect of omentin-1. Collectively, these results suggest that omentin-1 promotes the hOB proliferation via the PI3K/Akt signaling pathway. Several studies [39–41] have also uncovered that PI3K/Akt activation plays an essential role in osteoblast proliferation and recent data has demonstrated that PI3K/Akt plays a crucial role in osteoblastic differentiation of CVSMCs (calcifying vascular smooth muscle cells) induced by omentin-1 [17], findings that are in line with our present results.

In summary, our present study identified omentin-1 as an important regulator in human bone remodeling by promoting the hOB proliferation through the PI3K/Akt signaling pathway. These findings revealed the relationship between adipokines and bone metabolism and provided us a better understanding of the involved signaling mechanisms. Further investigation of omentin-1 and osteoblast may offer us a new target for osteoporotic prevention and treatment.
